# Tomographic Reconstruction of Neopterous Carboniferous Insect Nymphs

**DOI:** 10.1371/journal.pone.0045779

**Published:** 2012-09-25

**Authors:** Russell Garwood, Andrew Ross, Daniel Sotty, Dominique Chabard, Sylvain Charbonnier, Mark Sutton, Philip J. Withers

**Affiliations:** 1 School of Materials, The University of Manchester, Manchester, United Kingdom; 2 School of Earth, Atmospheric, and Environmental Sciences, The University of Manchester, Manchester, United Kingdom; 3 Department of Natural Sciences, National Museum of Scotland, Edinburgh, United Kingdom; 4 Muséum d'Histoire naturelle d'Autun, Autun, Bourgogne, France; 5 Département Histoire de la Terre, Muséum national d'Histoire naturelle, Paris, France; 6 Department of Earth Science and Engineering, Imperial College, London, United Kingdom; Ludwig-Maximilians-Universität München, Germany

## Abstract

Two new polyneopteran insect nymphs from the Montceau-les-Mines Lagerstätte of France are presented. Both are preserved in three dimensions, and are imaged with the aid of X-ray micro-tomography, allowing their morphology to be recovered in unprecedented detail. One–*Anebos phrixos* gen. et sp. nov.–is of uncertain affinities, and preserves portions of the antennae and eyes, coupled with a heavily spined habitus. The other is a roachoid with long antennae and chewing mouthparts very similar in form to the most generalized mandibulate mouthparts of extant orthopteroid insects. Computer reconstructions reveal limbs in both specimens, allowing identification of the segments and annulation in the tarsus, while poorly developed thoracic wing pads suggest both are young instars. This work describes the morphologically best-known Palaeozoic insect nymphs, allowing a better understanding of the juveniles’ palaeobiology and palaeoecology. We also consider the validity of evidence from Palaeozoic juvenile insects in wing origin theories. The study of juvenile Palaeozoic insects is currently a neglected field, yet these fossils provide direct evidence on the evolution of insect development. It is hoped this study will stimulate a renewed interest in such work.

## Introduction

During the Carboniferous most insects were hemimetabolous [1]. Rather than undergoing complete metamorphosis as holometabolous insects do, development of these insects is characterized by a series of nymphal stages similar in appearance to – but smaller than – adults [2]. Fossilised insect nymphs can provide important insights into the palaeoecology of their depositional environment and of ancient ecosystems; for example, ephemerid nymphs have been used to assess palaeoecology of Mesozoic lacustrine [3] and supratidal settings [4]. Furthermore, fossilised juveniles provide direct evidence of the evolution of insect developmental strategies such as complete metamorphosis [5], and hold a central role in Kukalová-Peck's wing origin theory [6], [7]. However, palaeoecological analyses for Palaeozoic deposits are difficult, in part because of the rarity of nymphal fossils (e.g. [8]). This is probably a taphonomic bias, the typically small, terrestrial and poorly sclerotized juveniles having a low preservation potential [9]; indeed, concentrations of juveniles are only found in sites of exceptional preservation. Analyses of known Palaeozoic juveniles are frequently hampered by reliability issues with the fossil data – critical insect fossils appear to have been compromised by intensive preparation [10]. A number of authors ([11], [12] and references therein) caution that the observations of Kukalová-Peck should be evaluated based on direct restudy of the specimens. Additional recurring problems with the study of Palaeozoic juveniles include a complex history of study, and identifying the adults to which juvenile taxa correspond [13]–[18].

Study of this material, whilst undeniably challenging, is nevertheless of great potential. For example, the hyperdiverse Endopterygota is a clade with ∼780,000 described species, which comprises more than 50% of the animal kingdom [19] and all members of which undergo complete metamorphosis. The earliest endopterygote insects are known from both Carboniferous body fossils [20], [21] and plant damage [22]. It is likely that renewed concerted study of juveniles from the Late Carboniferous – which has the earliest widespread insect fossil record – may reveal ‘larval’ stem-endopterygotes, and could thus inform our knowledge of endopterygote evolution, and in particular the evolution of their ontogeny.

The Late Carboniferous Montceau-les-Mines Lagerstätte of the Massif Central, France, is a site of exceptional preservation that has a surprising number of juvenile insects (almost half of the insects reported by Burnham [23] were immature). In common with a number of Late Carboniferous sites (e.g. Mazon Creek, USA [24], Coseley, UK [25]), the Montceau Lagerstätte preserves fossils as voids within siderite nodules [26]. Recent work has demonstrated the power of X-ray micro-tomography (µCT) in studying such fossils [27]–[29], revealing their morphology in full, and allowing better assessment of the palaeobiology, palaeoecology, and evolutionary relationships of such fossils. Here we report the µCT-based reconstruction of two juvenile insects from Montceau-les-Mines, discuss their palaeoecology, and highlight remaining difficulties in studying such taxa.

## Methods

### Material

Two fossils within small siderite nodules were scanned. The fossil MNHN.F.SOT086502 is a three-dimensional void, with some darker material – possibly phosphate – coating surfaces. The host nodule has split into four parts, one fracture between the dorsal and ventral surfaces revealing a coronal section, and a transverse fracture dividing the nymph across the metathorax. MNHN.F.SOT005630 is a void infilled with a white mineral, possibly kaolinite; the siderite nodule has split into three parts, the third of which is missing although it did not contain any fossil material. Little more than a cross section of the fossil is visible to the naked eye.

### X-ray Micro-tomography

Both fossils were scanned at the Natural History Museum, London on a Nikon Metrology HMX ST 225 CT scanner. MNHN.F.SOT086502 required a current/voltage of 185 µA/225 kV and MNHN.F.SOT005630 190 µA/225 kV. Both scans employed an unfiltered tungsten reflection target, and 3142 projections, providing a voxel size of 23 µm. Reconstructions – virtual models of the fossils – were created from the resulting tomograms using the custom SPIERS software suite [30]. For MNHN.F.SOT005630, all pixels darker than a user-defined grey-level were assumed to be fossil, through the creation of inverted linear threshold images. In addition to voids, MNHN.F.SOT086502 had partial pyrite infill, which comprised the lightest pixels in the tomogram, and thus a dual threshold was created. Artefacts were removed through manual cleaning, and regions of interest were defined for individual anatomical features, removing cracks from the models. The regions of interest were rendered as separate isosurfaces, and iterative improvements were made to their boundaries. For publication figures and animations, isosurfaces were ray-traced using the open source application *Blender* (blender.org). Models are included in the supporting information as animations (Video S1, S2), and also as downloadable virtual models in the form of zip-archived VAXML datasets (Model S1, S2; [30]; see also www.spiers-software.org).

### Nomenclatural Acts

The electronic version of this document does not represent a published work according to the International Code of Zoological Nomenclature (ICZN), and hence the nomenclatural acts contained in the electronic version are not available under that Code from the electronic edition. Therefore, a separate edition of this document was produced by a method that assures numerous identical and durable copies, and those copies were simultaneously obtainable (from the publication date noted on the first page of this article) for the purpose of providing a public and permanent scientific record, in accordance with Article 8.1 of the Code. The separate print-only edition is available on request from PLoS by sending a request to *PLoS ONE*, 1160 Battery Street, Suite 100, San Francisco, CA 94111, USA along with a check for $10 (to cover printing and postage) payable to “PLoS”.

In addition, this published work and the nomenclatural acts it contains have been registered in ZooBank, the proposed online registration system for the ICZN. The ZooBank LSIDs (Life Science Identifiers) can be resolved and the associated information viewed through any standard web browser by appending the LSID to the prefix “http://zoobank.org/”. The LSID for this publication is: urn:lsid:zoobank.org:pub:C629546C-37AB-4628-84AC-3E338CA0E86E.

## Results

### Systematic Palaeontology

Class Insecta Linnæus, 1758 [31]

Incertae familiae

Incerti ordinis

*Anebos* gen. nov.

*urn:lsid:zoobank.org:act:C39D14C6-A26C-4B68-9997-CD3A4260D5EA*

#### Etymology

Genus from Greek *anebos*, young, or beardless.

#### Diagnosis

As for type and only species.

#### Type species

*Anebos phrixos* sp. nov.

*Anebos phrixos* sp. nov.

urn:lsid:zoobank.org:act:18C692C2-0CC6-45CD-B4E5-321BE97972FF

#### Etymology

*Phrixos* is Greek for bristling; alluding to the defensive spines present in this juvenile insect.

#### Diagnosis

Heavily spined insect nymph, pronotum bearing six spines on lateral margin, opisthognathous head with prominent eyes and six spines on anterior margin, and abdominal segments with 3–4 spines on lateral margins and trilobate ventral surface. Terminal segmented cerci.

#### Material

Holotype specimen MNHN.F.SOT005630 (Collection Sotty 2, deposited in the Muséum d’histoire naturelle d’Autun, France, but belonging to the Muséum national d’Histoire naturelle, Paris, France).

#### Locality, horizon and age

Montceau-les-Mines Lagerstätte (Massif Central, France), Assise de Montceau Formation, Late Pennsylvanian ( = Late Stephanian in the European chronostratigraphic scale; [32]).

#### Description

**Figure 1:**
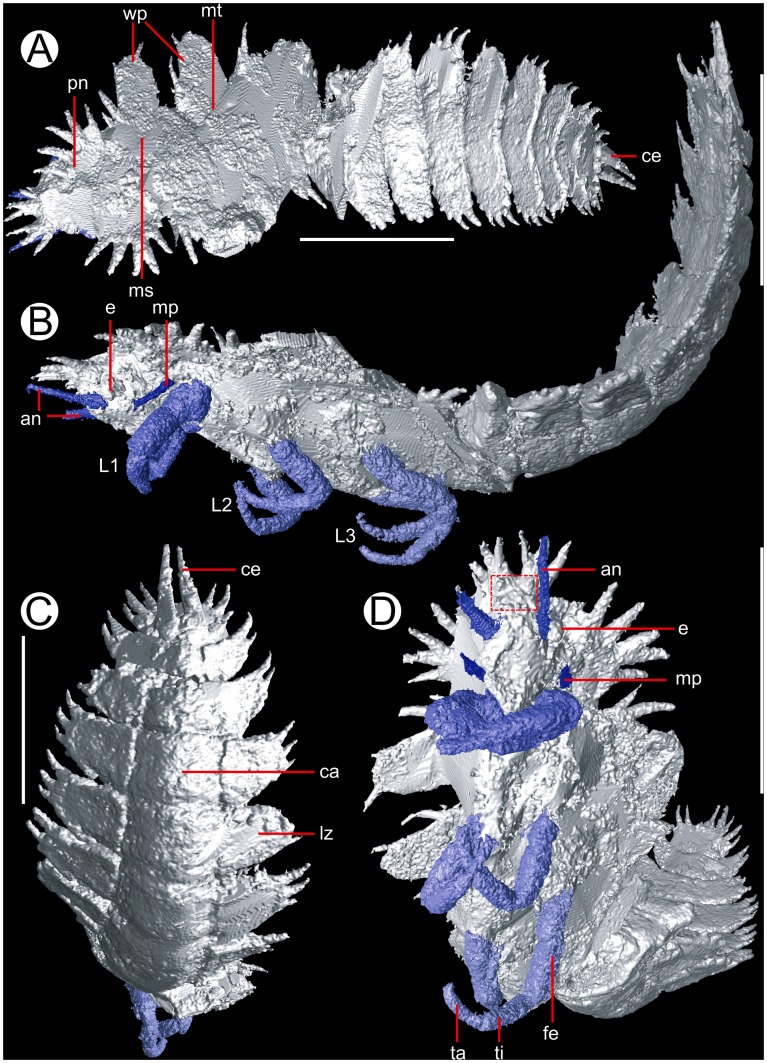
The insect nymph *Anebos phrixos* gen. et sp. nov. MNHN.F.SOT005630 from the Montceau-les-Mines Lagerstätte, France. A. Dorsal view, showing wingpads. B. Lateral aspect, of note is the orientation of the head and limbs. C. Ventral abdomen demonstrating trilobite underside. D. Anterior view, showing head, antennae and possible maxillary palps, in addition to leg segmentation. Abbreviations: an = antenna; ca = central axis; ce = cerci; e = eye; fe = femur; L1–3 = legs 1–3; lz = lateral zone; mp = maxillary palps (?); ms = mesonotum; mt = metanotum; pn = pronotum; ta = tarsus; ti = tibia; wp = wing pad. All scale bars equal 5 mm.

Insect nymph, 21.8 mm in length excluding anterior and posterior appendages, measured along the curved dorsal surface of the specimen. Heavily ornamented with dense spines on lateral margins for full length of body (Fig. 1A, Video S1). Strongly opisthognathous head tucked under pronotum (Fig. 1B), dorsal surface protrudes and bears six anterior spines. Ventral anterior of head slopes postero-ventrally and bears an array of smaller tubercles demarking a square (outlined with a red dotted line in Fig. 1D). Immediately posterior to this attach forward-facing antennae; segmentation of antennae not clear beyond larger basal segment, likely filiform. Right antenna truncated after 0.8 mm, left after 1.59 mm. Ventral and posterior to antennal attachment is prominent eye (Fig. 1D), details poorly preserved, but appear tubercular, protruding ∼0.5 mm from the lateral body wall. Mouthparts not well preserved, posteriorly directed and triangular in form from below, terminating between the first pair of legs. Pronotum (4.2 mm in length) narrower than mesonotum and metanotum, with a fan of four lateral spines, one bifurcating.

Appendages immediately anterior to the forelegs difficult to interpret, as no clear segmentation preserved. Interpretation as exopods of a (biramous) prothoracic limb cannot be excluded, but such structutes would be unexpected [33], especially in a single limb pair. Maxillary palp interpretation more parsimonious; limb origin lies near mouthparts, although posteriorly-directed head results in close proximity of mouthparts and limb bases, and exact origin cannot be traced.

Limbs well resolved. Prothorax bears the first pair of legs (Fig.; 1D), comprising short, rounded coxa (0.5 mm), a small trochanter, then long femur (1.69 mm). Leg bends at femur-tibia joint; this bend is interpreted as a 'death posture' (i.e. not a natural *in vivo* condition). Tibia relatively short (1.1 mm), and limb terminates with annulated tarsus (1.1 mm). Annuli not clear, but curvature in this region indicative of pseudosegmentation. Limbs are short and robust. Pair of smaller 'appendages' immediately anterior to first limbs are present (see Discussion). Despite apparent origin immediately anterior to the first pair of limbs there is little evidence for attachment in this position. Like antennae show little evidence of segmentation, but curvature suggests that segmentation or annulation was present.

Mesonotum (2.2 mm in length) bears two spines (Fig. 1A), anterior to a narrow wing pad, with wide attachment to the body and lateral spine at apex. Wing pad is posteriorly directed but with no obvious point of curvature, and while (laterally) wide is shorter than typical for insect nymphs (c.f. cockroach nymph below). Only preserved on one side. Mesothorax bears second pair of legs, coxa and trochanter less well-preserved than first pair, but otherwise well-resolved. Limbs more gracile than prothoracic pair, and terminate in a pretarsal claw. Body here relatively deep but narrow, and skewed to right side suggestive of post-mortem distortion.

Metanotum (∼3.7 mm) bears posteriormost wing pad, similar in size and shape to that of mesonotum, but with spine on wingpad at anterior (leading edge) of the pad rather than apex (Fig. 1A). Terminal triple spike at posterior of segment, behind wing pad. Wing pad only preserved on right side. Metathoracic legs similar in shape to those of mesothorax, but slightly longer. Annulation in tarsus clearest here. In contrast to forelegs, mesothoracic and metathoracic appear to attach posteriorly directed.

Thorax-abdomen boundary poorly preserved. Abdomen preserved dorsally recurved, with ten abdominal segments (Fig. 1B). Each bears three prominent spines on the lateral border, larger segments have a small fourth on their anterior margin. Spines increasingly posteriorly directed towards the end of the abdomen, except the tenth which is only ventrally expressed with a pair of posteriorly directed spine-like cerci possessing no visible segmentation. Ventral surface of abdominal segments are trilobate (Fig. 1C), with a semi-circular central lobe and then wedge-shaped lateral zones. The central axis decreases in width posteriorly while the lateral zones do so to a lesser degree.

Superorder Dictyoptera

Unnamed juvenile

#### Material

MNHN.F.SOT086502 (Collection Sotty 2, deposited in the Muséum d’histoire naturelle d’Autun, France, but belonging to the Muséum national d’Histoire naturelle, Paris, France).

#### Locality, horizon and age

Montceau-les-Mines Lagerstätte (Massif Central, France), Assise de Montceau Formation, Late Pennsylvanian ( = Late Stephanian in the European chronostratigraphic scale; [32]).

#### Description

**Figure 2:**
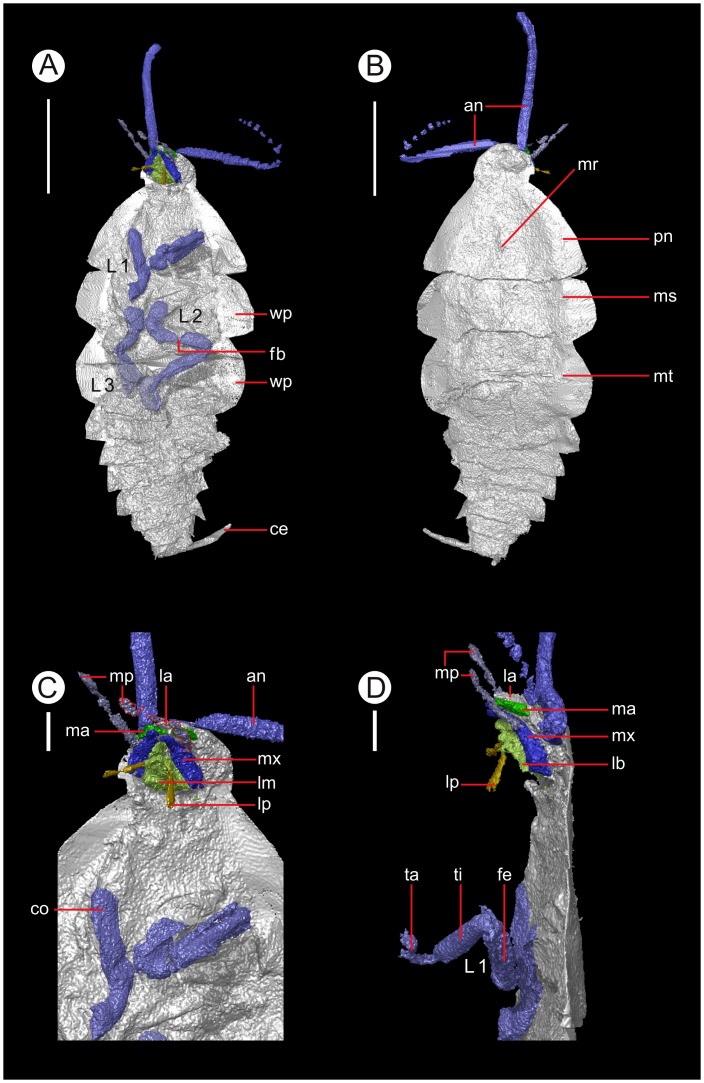
The roachoid insect nymph described herein: MNHN.F.SOT086502 from the Montceau-les-Mines Lagerstätte, France. A. Ventral view, showing limbs, head appendages and cerci. B. Dorsal view, showing wing pads. C. Ventral head showing mouthparts. D. Lateral view with leg segmentation and mouthparts labelled, antennae removed. Abbreviations: an = antenna; ce = cerci; co = coxa; e = eye; fe = femur; fb = femur break, reconstruction artefact resulting from the switch between pyrite infill and void; L1–3 = legs 1–3; la = labrum; lm = labium; lp = labial palp; ma = mandible; mp = maxillary palp; mr = median ridge; ms = mesonotum; mt = metanotum; mx = maxilla; pn = pronotum; ta = tarsus; ti = tibia; wp = wing pads. Scale bars: A,B = 5 mm; C,D = 1 mm.

**Figure 3:**
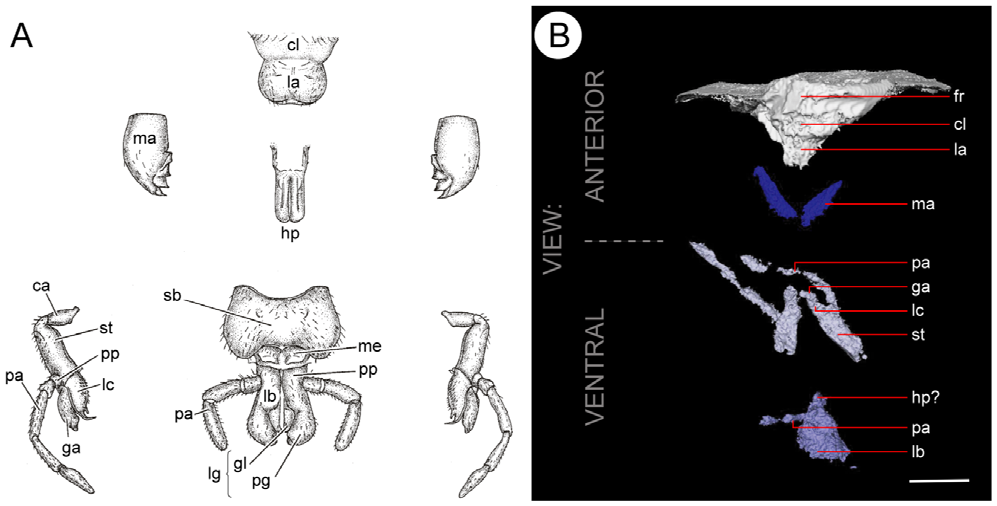
Insect mouthparts. A. Those of a typical biting-chewing insect, from Brusca & Brusca [77]. B. B. The mouthparts of the roachoid nymph MNHN.F.SOT086502 revealed by µCT. Abbreviations: ca = cardo; cl = clypeus; fr = frons; ga = galea; gl = glossa; hp? = possible hypopharynx; la = labrum; lb = labium; lc  = lacinia; lg = lingul; ma = mandible, me = mentum; pa = palp; pp = palpiger; sb = submentum; st = stipes. Scale bar in B = 1 mm.

A small roachoid nymph, 21.1 mm in length excluding appendages, semicircular head at anterior (Fig. 2B, Video S2). Two antennae attach at the anterior cephalic margin, and comprise a large number of small segments – the right is prematurely truncated, while it is possible the left is complete (8.5 mm), narrowing towards its apparent termination. Antennae had a minimum of 23 frustal/situliform segments in life. One is held parallel to the long axis of the body, the other perpendicular to this, with a bend midway (Fig. 2A,B). Eyes not resolved. The ventral head preserves the mouthparts in their entirety (Fig. 3B). The anterior cephalic margin appears to possess a frons, clypeus and then a triangular labrum (the latter dorso-ventrally 0.5 mm), immediately anterior to well-preserved, slender mandibles, one displaying both condyles. Posterior to these are the maxillae, with stipes, lacinia and galea present on both sides. As a result of the required arbitrary termination of limbs when separating them from the body during the computer reconstruction, the cardo could not be identified. The palps possessed a palpiger and a minimum of four segments, both are lengthy (2.3 mm), outstretched and skewed towards the right, but not very well resolved (Fig. 2C,D). Posteriormost is the labium, with broad attachment at the base of a submentum. Mentum not distinguishable, but prementum can be discerned between the labial palps. These comprise a minimum of two segments, and just anterior to the labium is an elongate structure with triangular cross section; this is likely the hypopharynx.

Posterior to the mouthparts is a broad (8.3 mm at widest point) pronotum, 4.9 mm in length (Fig. 2B) with gently curved lateral margins and a median ridge. The left foreleg is one of the two complete limbs, and comprises a small coxa (0.9 mm) and trochanter, then longer femur (2.5 mm). The femur is somewhat flattened in cross section and possesses longitudinal ridges. Tibia a similar length (2.3 mm), and more circular in section proximally, but distally has a flattened dorsal surface. The five segmented tarsus comprises short four short tarsomeres and a long terminal pseudosegment, with strong curvature in the first three. Limb terminates with a pretarsal claw. The left limb truncates mid-femur. The mesonotum is somewhat shorter (3.4 mm) but otherwise similar in shape. Wing pads possess a broad attachment to the tergite, and have a gently curved lateral margin. The mesothorax bears the other complete limb (Fig. 2A), identical in form to the previously described appendage but more posteriorly directed. Its opposite terminates mid-femur. Metanotum is the longest thoracic tergite (4.1 mm) with similarly large wing pads. These have a more subtle posterior curve than the mesothoracic pads. Poorly preserved metathoracic limbs truncate post-coxa.

Abdomen well-resolved, although the lateral margins are difficult to differentiate from the crack along which the nodule was split, making them somewhat subjective in the model (Fig. 2A,B). The nine abdominal segments shorten slightly posteriorly (first: 1.3 mm in length, penultimate 1.1 mm), terminal segment small and situated between the cerci. Cerci lack discernible segmentation; one directed laterally and probably complete, the other dorsally directed and prematurely truncated. Abdominal segments narrow posteriorly, each being associated with a lobe in the lateral margin of the abdomen. The ventral surface of some areas appears distorted, with ventral plates detached (e.g. right edge of abdominal segments 4 & 5) – coupled with the poorly preserved legs suggesting post-mortem decay. The body is fairly flat; the retained three-dimensionality of the limbs suggests that this is not a taphonomic effect.

### Remarks

The two nymphs described here with the aid of µCT are remarkably disparate in form. *Anebos phrixos* is heavily spinose on the head, thorax and abdomen, making it unique amongst Palaeozoic insects. Although some groups are known to have a spinose thorax (e.g. Palaeodictyoptera: *Notorachis*; Megasecoptera: *Mischoptera*; and members of the family Geraridae) they lack spines on the head and abdomen [34]. As this insect is likely to be polyneopteran (see discussion), the adult will be similar in habitus. As such we believe that describing this juvenile as a new genus and species is justified. The second nymph is typical of Carboniferous roachoid (‘blattopteran’) juveniles in the form of the pronotum, wing pads, and abdomen (e.g. see [35]). However, many of these are already named, and could be the nymphs of named adults. Thus we believe there is no reason to name this specimen.

## Discussion

### Other Montceau-les-Mines Juveniles

Adult insects from Montceau-les-Mines comprise a ‘typical’ Carboniferous insect fauna, belonging to extinct palaeopteran orders (e.g. Palaeodictyoptera, Megasecoptera), stem-lineages of extant groups (e.g. cockroaches, mayflies, grasshoppers and crickets), and taxa of uncertain affinities (e.g. miomopterans). Less is known of the juveniles in this fauna, although they are abundant: Burnham [23] reports that of the 110 insects found, 49 are immature (see also [36]). ‘Cockroach nymphs’ have been reported in passing from the site [23] and the remaining taxa have been split into two broad categories (descriptions cited as in preparation by Burnham [23]). Members of the first are referred to as ‘megasecopteroid’ nymphs because they resemble nymphs of the extinct palaeodictyopteroid order Megasecoptera [37], with narrow, elongate abdomens, and thickened, leathery wing buds that curve away from the body. The second group are referred to as ‘ephemeropteroid’ nymphs because they resemble ephemeropteran (mayfly) nymphs [38], possessing a broader abdomen with paired lateral ‘winglets’ on each abdominal segment, and developing membranous wings with clear venation. These are smaller than the 30 mm 'megasecopteroid' nymphs. Both may have been aquatic.

Neither of the nymphs described here fit into either of these broad categories of Montceau-les-Mines juveniles. They lack a long abdomen and posteriorly directed wing pads [23] – indeed neither resembles any known Carboniferous palaeopteran juveniles. However, both are united by a small size and poorly developed wing pads, which is indicative of young instars (in hemimetabolous development the wings increase in size with each moult: [2]). Neither exhibits evidence for a posited ancestral state of seven segmented tarsi [39]; instead, both appear to possess five tarsomeres and pretarsal claw, in keeping with the assumption [11], [40] that this condition is plesiomorphic to the Pterygota (winged insects).

### Aquatic or Terrestrial?

No close modern analogues for *A. phrixos* are known, but its morphology suggests that it was terrestrial. The lateral extensions of the abdomen – situated where gills would occur in a naiad – are spinose, with broad attachments and small surface area. Like those of the pro- and mesonotum they were probably defensive. In contrast, as reviewed by Bitsch [41], the gills of extant naiads possess either an articulated attachment to a basal lobe with associated musculature (Ephemeroptera, damselflies) or are simpler, tubular evaginations from the pleural membrane with associated musculature arising from an adjacent tergal plate (other odonates). In Plecoptera they are either filamentous, subsegmented tracheal gills with associated musculature (family Eustheniidae) or are simpler branched processes that are variously located on the head, thorax, and first abdominal segments. Even the simplest of these structures bear little similarity to the spines of *A. phrixos*.

The roachoid is similar in habitus to extant roach nymphs, which are – with very few exceptions – terrestrial. Rare extant amphibious and quasi-aquatic cockroaches show few external morphological adaptations [42]. While a terrestrial mode-of-life is the most parsimonious interpretation, a partially aquatic lifestyle cannot be excluded.

While the taphonomic loss of gills – composed of labile tissues that decay rapidly – is possible in either fossil, we think this unlikely. Other labile structures are preserved in these fossils, such as eyes and easily-disarticulated mouthparts. The first stage of insect decay in experimental studies is the expansion of internal tissues, stretching the arthroidal membrane between abdominal segments [43]. No such expansion is present in either nymph fossil, confirming a very low degree of pre-fossilisation decay. Gills are preserved in the co-occurring Montceau ephemeropteran nymphs [23], and also typically in other sites of exceptional preservation where aquatic insects are found [44], [45]. Even if gills have been lost through decay we would expect to see attachment structures; these are absent in both fossils.

Thus, multiple lines of evidence support the suggestion these nymphs were terrestrial. Gills were once inferred to have been almost universal in Palaeozoic pterygote nymphs, under the hypothesis that Pterygota possess plesiomorphically aquatic juveniles [7], [46]. This hypothesis arose, in part, because aquatic nymphs are present in lineages considered the most basal amongst the winged insects – i.e. the ‘palaeopteran’ orders Odonata (dragon- and damselflies) and Ephemeroptera (mayflies), and in the Plecoptera (stoneflies) which some authors consider basal in the Neoptera [47] (neopteran insects can fold their wings over the abdomen, an ability the palaeopteran orders plesiomorphically lack). However, support for these relationships remains equivocal; palaeopteran and polyneopteran relationships (including those of the Plecoptera) being particularly problematic [48]. Furthermore – and more to the point – recent evidence suggests that aquatic juveniles evolved independently in each group [2]. For example, abdominal gills are considered doubtful in the plecopteran ground pattern [49], whilst the location on the body and structure of gills suggest convergent origins in the Ephemeroptera, Odonata, and Plecoptera [1] (for further discussion see Bitsch [41]). If juvenile pterygotes were plesiomorphically aquatic, a clear taphonomic bias would exist favouring their preservation. Such fossils are rare prior to the Triassic, suggesting a limited number of groups possessed naiads prior to the Mesozoic [50]–[52]. Current evidence hence supports a secondarily aquatic model for pterygote juveniles, which would have evolved from fully terrestrialised ancestors [53], [54]. The fact that these Carboniferous Neoptera were likely terrestrial is congruent with this hypothesis: both would have been washed from vegetated areas into the lacustrine-deltaic depositional setting that the Montceau-les-Mines deposits represent [55].

### Wing Origins and Palaeozoic Juveniles

Evidence from Palaeozoic juveniles is used extensively by Kukalová-Peck, to support the exite-wing theory. For example, she stated that “Paleozoic nymphs of primitive Neoptera and of all Palaeoptera […] including Ephemerida, have articulated wing cases” [7] and “primitive articulation and mobility of nymphal wings and the ‘pleural appendage’ theory of wing origin are two sides of the same coin” [6]. The articulated nature of these wings is used by Kukalová-Peck – through a recapitulation model – to support the idea that wings are plesiomorphically free lateral structures, as would be expected from the exite-wing theory (in contrast to the fixed paranotal lobes of alternative theories).

The wing pads described here do not possess an articulation with the thorax, or any evidence of mobility – both have a simple and broad attachment. While this appears to support Wootton's view [17] that articulation is not as universal as previously suggested (contra [56]) we do not believe that these nymphs – or other Palaeozoic juveniles – can, at present, inform debates regarding the origin of insect wings. It is likely that Carboniferous nymphs post-date the origin of wings by tens of millions of years, and the same is true of fossils used to support the exite-wing theory which are Carboniferous or younger in age [6], [7], [57], [58]. In contrast *Rhyniognatha hirsti* – an insect argued to have wings by Engel & Grimaldi [59] – is ∼411 million years in age [59], while molecular estimates place this split at 455 Ma [60]. Besides issues regarding the reliability of the raw data (see discussion in [10], [12], [61]), this age relationship makes models of phylogeny an integral aspect of this debate. With limited temporal evidence, the plesiomorphic condition of pterygotes can only be assessed in the light of a stable phylogeny within which to place observations from fossil taxa. Without this, symplesiomorphies supporting any wing origin theory could as easily prove to be synapomorphies. If the nymphal wing articulation used in support of the wing-exite hypothesis [6], [58] is found in the ‘palaeopteran’ orders, this could only be considered a symplesiomorphy if the Palaeoptera were not monophyletic – which is currently an open question [62]. Furthermore the identification of a ‘primitive’ Neopteran relies upon not only a phylogeny of the Polyneoptera (also currently lacking [48]), but the ability to place the juveniles reliably within an order. Without a stable phylogeny, earlier taxa or increased understanding of the evolution of insect ontogeny, using the morphology of Carboniferous juveniles to support wing origin theories is fraught with difficulty.

### Anebos Phrixos

The difficulty of identifying the adult relatives of Palaeozoic nymphs is clear from the literature [13]–[18]. Nevertheless, speculation is possible. The opisthognathous condition of *Anebos phrixos* obscures many of the details of the mouthparts. However, no terminal structures that would be indicative of a haustellate arrangement are visible protruding from between the limbs. This rules out extinct palaeopteroids with a haustellate arrangement (Diaphanopterodea, Paleodictyoptera, Megasecoptera, and Permothemistida [37], [56]), and also the hemipteroid insects [2]. A placement within the odonatoid clade is also unlikely; a lack of pronotal lobes excludes assignment to the Geroptera and all known odanotoid juveniles have predacious aquatic naiads. It is uncertain, however, whether Palaeozoic odonatoid nymphs were terrestrial or aquatic; a (semi) aquatic mode of life has recently been reported in Carboniferous protodonatoids [54], but such findings should be assessed with caution for reasons already elaborated. *A. phrixos* lacks the labial mask, large compound eyes and gills diagnostic of such taxa. The presence of wing pads preclude placement within the Endopterygota. Accordingly the most likely affinity for this nymph is within the stem-Orthoptera.

We believe the spined habitus of *A. phrixos* was a defensive adaptation; without flight to escape danger, in Carboniferous Coal Forests awash with potential predators, nymphs were at great risk [63]. Heavy spination would make the nymph less palatable – a fact reflected also in contemporaneous, heavily spinose Myriapoda (e.g. euphoberiid diplopods). At this time amphibian predators lacked differentiated teeth and were likely inertial feeders with little mastication prior to swallowing [64]. Vertebrate predators found at Montceau-les-Mines include aïstopods [65] and branchiosaurs [66]. Carboniferous arachnid predators included scorpions [67] and trigonotarbids [68], while predacious insects also existed. These included aerial hunters such as griffenflies (Protodonata/Meganisoptera) [69] and possibly mayflies (Ephemeroptera) [70], while the abundant stem-Orthoptera could have included ground-based predators [71], [72], and contemporaneous stem-Mantodea [73] may have shared the diet of their crown-group descendants. The archaeorthopteran *Ctenoptilus elongatus* (Brongniart, 1893) [74] possessed tibial and femoral spines on the fore- and midlegs, and lateral extensions on selected foreleg tarsomeres [71]. As posited adaptations towards predation, these features could have helped the handling of spined juveniles such as *A. phrixos*. A lack of haustellate mouthparts suggests the nymph employed a form of feeding other than piercing-and-sucking [70]. Typically, opisthognathous mandibulate mouthparts – seen in some beetles – are employed for detritivory (C. Labandeira, pers. comm.), making this a likely mode for *A. phrixos*.

### Roachoid

The biting mouthparts of this nymph rule out many palaeopteran affinities: the Palaeodictyopteroidea had sucking mouthparts [37], [56], and on the basis of extant taxa we would expect odonatoid naiads to possess labial masks, large compound eyes and be aquatic. The nymph lacks caudal filaments, and is thus not ephemeropteran [38]. The mouthparts appear polyneopteran, as discussed in detail below. The flattened habitus is similar to that of modern cockroach nymphs, with a large pronotum, well developed cerci, and long antennae. The fossil resembles published Carboniferous roachoid nymphs [46], [75]. As such, it seems likely this is a nymph of the Blattoptera.

The high resolution and detail recovered for this nymph's mouthparts is of note – not only are these amongst the best resolved Carboniferous insect mouthparts, but the labrum, the mandibles for processing food, the hypopharynx to aid swallowing, and maxillae and labium are all resolved (Fig. 3). Both of the latter possess palps that (by comparison to modern forms) probably aided the manipulation and chemoreception of food [76]. They are essentially the same as the generalized mandibulate mouthparts seen in more basal orthopteroid insects. For example, they possess mandibles lacking specialized processes, their maxillary palpi possess five articles and show little specialisation, the three-segmented labial palpi are similarly generalized, and their hypopharynx lacks specialized epipharyngeal structures. These same structures are found in modern cockroaches and other generalist feeders. With little evidence of specialisation, the mouthparts of the nymph point towards a generalist diet. Much like modern forest roach nymphs, they could have eaten decaying and rotting matter on the forest floor. Highly developed and mobile antennae, again like those of modern roaches, suggest a well-developed sensory apparatus. Modern roach nymphs forage at night [42] – it is possible the same applies to this species, which does not possess well-developed eyes (none are discernible in either the scan or hand specimen). The flattened nature of the nymph likely allowed it to negotiate and live in the leaf litter. It would have provided defence, allowing it to flatten itself against surfaces without causing shadows [46], made it more difficult to pick up, and facilitated a cryptic lifestyle, sheltering in narrow crevices and under tree barks and logs [42].

## Supporting Information

Model S1 **VAXML model of the nymph** ***Anebos phrixos*** **gen et sp. nov.**
(ZIP)

Model S2 **VAXML model of the roachoid nymph.** For viewing, models should be unzipped and a suitable VAXML viewing package installed; if SPIERS is used to view the datasets, the user need only double-clicking on the.vaxml file. Both models have been processed to reduce triangle counts with a 'quadric' fidelity-reduction algorithm (built into SPIERS), but low-performance systems may nonetheless struggle to render them.
(ZIP)

Video S1 **Animation showing the reconstruction of the nymph** ***Anebos phrixos*** **gen et sp. nov.**
(AVI)

Video S2 **Animation showing the reconstruction of the roachoid nymph.**
(AVI)
